# Aspirin Use and Risk of Pancreatic Ductal Adenocarcinoma: A Large Case-Control Study

**DOI:** 10.34172/aim.2023.28

**Published:** 2023-04-01

**Authors:** Zahra Momayez Sanat, Sahar Masoudi, Seidamir Pasha Tabaeian, Maryam Jameh Shorani, Majid Soruri, Akram Porshams

**Affiliations:** ^1^Liver and Pancreatobiliary Diseases Research Center, Digestive Diseases Research Institute, Tehran University of Medical Sciences, Tehran, Iran; ^2^Department of Internal Medicine, School of Medicine, Hazrat-e Rasool General Hospital, Iran University of Medical Sciences, Tehran, Iran; ^3^Department of Internal Medicine, School of Medicine, Zanjan University of Medical Sciences, Zanjan, Iran; ^4^Digestive Oncology Research Center, Digestive Diseases Research Institute, Tehran University of Medical Sciences, Tehran, Iran; ^5^Gastrointestinal and Liver Diseases Research Center, Guilan University of Medical Sciences, Rasht, Iran

**Keywords:** Aspirin, Case-control study, Pancreatic cancer, Pancreatic ductal adenocarcinoma

## Abstract

**Background:**

Pancreatic ductal adenocarcinoma (PDAC) is one of the deadliest cancers, with a five-year survival rate of approximately 5%. The incidence and mortality rates of PDAC are increasing, and the results of medical treatments remain unsatisfactory. Some conflicting evidence suggests that aspirin intake may reduce the risk of PDAC. This study aimed to evaluate the association between regular low-dose aspirin use (80-mg aspirin tablets, 5-7 tablets/week) and the risk of PDAC.

**Methods:**

This prospective, hospital-based, case-control study was performed on 470 PDAC patients (case group) and 526 sex and age-matched controls, in Tehran, Iran from 2011 to 2018. The participants were interviewed regarding the patterns of aspirin use. Data are expressed as mean±SD or frequency and percentage as appropriate. Differences in frequency between the case and control groups were evaluated based on the analysis of the contingency table (χ^2^ test and Fisher’s exact test). Propensity score models were designed to calculate odds ratios (OR) and 95% confidence intervals (95% CIs) for PDAC with respect to aspirin use, adjusted for age, sex, smoking status, opium use, diabetes mellitus, place of residence, and family history of cancer in first-degree relatives.

**Results:**

About 60% of PDAC patients were male in this study. Also, 25.2% of PDAC patients had a family history of cancer in one of their first-degree relatives, 21.99% were smokers, 13.9% were opium users, and 11.7% had a history of diabetes. Aspirin was used by 22.77% of PDAC patients and 18.25% of the controls. Ever aspirin use (OR: 1.01, 95% CI: 0.89 – 1.14) was not associated with PDAC.

**Conclusion:**

Overall, aspirin use was not associated with a reduced risk of PDAC.

## Introduction

 Pancreatic ductal adenocarcinoma (PDAC) is a deadly cancer, with rising incidence and mortality rates worldwide.^1^ In 2017, nearly 441 000 deaths caused by PDAC were reported worldwide.^1^ There was a 2.3-fold increase in the global incidence and mortality of PDAC from 1990 to 2017, indicating both the aging and growth of the population and the increased prevalence of obesity and diabetes, which are two main risk factors for PDAC.^2-4^ The five-year survival rate of PDAC has slightly improved in the past four decades from 3% in 1970 to 5% today.^5,6^ PDAC is diagnosed frequently at an advanced stage when surgical resection is not possible, and chemoradiation is not effective enough.^6^ Considering the slow progress in the management of PDAC burden, efforts are being made today to find early markers and introduce potential chemo-preventive agents.

 Aspirin is a non-steroidal anti-inflammatory drug (NSAID), primarily used for prevention and treatment of cardiovascular disease.^7^ However, its long-term use has been associated with a reduction in the overall cancer risk due to its antioxidant and anti-inflammatory properties and inhibition of cyclooxygenase-2 (COX-2) pathway with increased expression in PDAC.^8-10^ According to laboratory studies, inhibition of COX-2 activity may be an effective preventive approach against PDAC.^11,12^ Nevertheless, the association between aspirin use and the risk of PDAC is inconsistent in clinical studies.^13-16^ Therefore, the present study aimed to evaluate the association between low-dose aspirin use (80-mg aspirin tablets, 5-7 tablets per week) and the risk of PDAC.

## Materials and Methods

 The recruitment methods of case and control groups have been extensively explained in the literature and briefly described here.^17^ The case group (patients with pathology-proven PDAC) and the control group (individuals with similar referral patterns and gastrointestinal motility disorders or biliary stone disease with a normal pancreas and no other cancers or organ failure), matched by sex and age, were recruited from Shariati Hospital (a tertiary referral hospital) in Tehran, Iran, between December 2011 and January 2018.

 Individuals suspected of having pancreatic cancer were invited to participate in this case-control study. Upon enrolment, written informed consent was obtained from the participants. Next, bio-sample collection and endoscopic ultrasonography were performed for the patients. If a mass or cystic lesion was detected, fine-needle aspiration was performed. The obtained samples were then reviewed by two expert pathologists, who were blinded to the questionnaire data. All participants with histologically confirmed PDAC were included in the study. For data collection, a valid and reliable, structured questionnaire was used by a few trained interviewers before performing endoscopic ultrasonography.^18^ The participants were asked regarding regular use of aspirin (80-mg aspirin tablets, 5-7 tablets/week). For each episode of use, the name of the drug, date of drug use onset, and duration and frequency of drug use were recorded.

 Data are expressed as mean ± SD or frequency and percentage as appropriate. Differences in frequency between the case and control groups were evaluated by analysis of contingency table (Fisher’s exact test and χ^2^ test). Since case-control studies have inevitable weaknesses, we attempted to reduce bias by using propensity match scores to achieve a balance of covariates, which would allow for a more robust estimation of the treatment effect.^19,20^ We considered age, sex, smoking status, opium use, diabetes mellitus, place of residence, and family history of cancer in first-degree relatives as confounding factors.^17,21,22^ Models were run separately for those who ever used aspirin, for those who used it for more than 1 year, for those who used it for more than 5 years, and for those who used it for more than 10 years. Odds ratios (ORs) and 95% confidence intervals (CIs) were estimated using logistic regression models. All statistical analyses were performed in STATA version 11 (STATA Corp., College Station, TX, USA). A *P* value less than 0.05 was considered significant. It has been previously shown that cigarette smoking, opium use ( > 1 year), long-term diabetes mellitus ( > 2 years), and a family history of cancer in first-degree relatives with no obesity were associated with an increased risk of PDAC development in our population.^17,21,22^

## Results

 A total of 470 new incident cases of histologically confirmed PDAC and 526 hospital controls were enrolled in this study. The descriptive characteristics of PDAC cases and controls are presented in Table 1. Nearly 60% of the patients were male. The mean age of the patients was 64.10 ± 11.55 years, and 80% of them were urban inhabitants. Approximately 25% of patients had a history of cancer in their first-degree relatives, 21.99% smoked, and 13.86% used opium. Moreover, 11.75% of the patients had a history of diabetes for more than 2 years. The PDAC patients were more likely to be smokers, opium users, and rural inhabitants. Also, the case group had a higher frequency of a previous diagnosis of diabetes mellitus and a family history of cancer in their first-degree relatives compared to the controls (Table 1).

**Table 1:** Descriptive Characteristics of Pancreatic Cancer Cases and Controls

**Participants Characteristics**	**Total (n=996)**	**Cases (n=470)**	**Controls (n=526)**	***P*** ** Value**
Age (years) mean ± SD	63.38 ± 12.47	64.10 ± 11.55	62.74 ± 13.22	0.072
BMI (kg/m^2^) ± SD	26.29 ± 5.93	26.18 ± 5.05	26.73 ± 5.89	0.147
Gender				
Male	597 (59.94%)	284 (60.43%)	313 (59.51%)	0.767
Female	399 (40.06%)	186 (39.57%)	213 (40.49%)	
Education				
Illiterate	379 (38.05%)	180 (38.30%)	199 (37.83%)	0.513
Less or high school graduate	495 (49.70%)	227 (48.30%)	268 (50.95%)	
Advanced degree	122 (12.25%)	63 (13.40%)	59 (11.22%)	
Residence				
Rural	155 (15.64%)	92 (19.66%)	63 (12.05%)	0.001^*^
Urban	836 (84.36%)	376 (80.34%)	460 (87.95%)	
Marital Status				
Single	19 (1.91)	5 (1.06%)	14 (2.66%)	0.154
Married	795 (79.82%)	382 (81.28%)	413 (78.52%)	
Divorced or widowed	182 (18.27%)	83 (17.66%)	99 (18.82%)	
Family history of any cancer (first degree)				
Yes	251 (25.20%)	138 (29.36%)	113 (21.48%)	0.004^*^
No	745 (74.80%)	332 (70.64%)	413 (78.52%)	
Diabetes mellitus history ( > 2 years)				
Yes	117 (11.75%)	70 (14.89**%**)	47 (8.94**%**)	0.004^*^
No	879 (88.25%)	400 (85.11**%**)	479 (91.06**%**)	
Smoking status				
Current	219 (21.99%)	123 (26.17%)	96 (18.25%)	0.011
Quit more than 5 y	104 (10.44%)	46 (9.79%)	58 (11.03%)	
Never	673 (67.57%)	301 (64.04%)	372 (70.72%)	
Opium use				
More than 1 year	138 (13.86%)	77 (16.38%)	61 (11.60%)	0.029^*^
Never	858 (86.14%)	393 (83.62%)	465 (88.40%)	

*P< 0.05 is significant.

 The results regarding the aspirin intake of the study population are presented in Tables 2 and 3. Low-dose aspirin intake was not associated with a reduced risk of PDAC (adjusted OR [aOR]: 1.01, 95% CI: 0.89–1.14).

**Table 2:** Description of Aspirin Use and Duration among Cases and Controls

**Duration of use**	**Total**	**Cases **	**Controls **	***P*** ** Value**
Ever Aspirin user	203 (20.38%)	107 (22.77%)	96 (18.25%)	0.077
Aspirin user more than 1 year	154 (15.46%)	82 (17.45%)	72 (13.69%)	0.101
Aspirin user more than 5 year	85 (8.53%)	48 (10.21%)	37 (7.03%)	0.073
Aspirin user more than 10 year	28 (2.83%)	17 (3.62%)	11 (2.11%)	0.150

**Table 3:** Pancreatic Cancer Odds Ratios According to Duration of Aspirin Use

**Variables**	aOR^*^	**95% CI**
Aspirin user Ever	1.01	0.89–1.14
Aspirin user, > 1 year	0.91	0.82–1.01
Aspirin user, > 5 year	0.93	0.84–1.01
Aspirin user, > 10 year	0.95	0.65–1.38

aOR, adjusted odds ratio. ^*^Propensity score matching by age, gender, smoking, opium use, diabetes mellitus, residence and family history of any cancer in first-degree relatives.

## Discussion

 PDAC accounted for 466 000 deaths in 2020, and it was the seventh leading cause of cancer-related mortality in both males and females.^23^ The incidence, prevalence, and mortality of PDAC have increased by 55%, 63%, and 53%, respectively, in the last 25 years.^24^ Both the incidence and mortality rates of PDAC have been either stable or increased globally, suggesting the increasing prevalence of obesity and diabetes mellitus, although advances in diagnostic and cancer registration modalities may have been also effective in some countries.^3,22,25^ It has been proposed that the mortality rates of PDAC surpass those of breast cancer. This type of cancer may become the third leading cause of cancer death following lung and colorectal cancers in 28 European countries by 2025.^26^

 With the translation of emerging technologies to early diagnostic tools and more effective therapies for PDAC, it is crucial to prevent PDAC. Aspirin, with antioxidant and anti-inflammatory properties and inhibition of COX-2 pathway, is considered a chemoprophylaxis agent for PDAC management^8-10^; however, the association between aspirin use and the risk of PDAC is inconsistent in clinical studies.^13-16^ The results of the present study on the ineffectiveness of low-dose aspirin in reducing the incidence of PDAC are consistent with the results of most relevant clinical studies. In this regard, in a clinical trial on women, Cook et al showed that daily low-dose aspirin use (100 mg) over 10 years did not reduce the PDAC risk.^27^ Moreover, Choi et al evaluated the association between the use of aspirin and PDAC in a nested case-control study on a 12-year nationwide Korean cohort.^28^ They recruited 827 PDAC patients and 4,135 matched controls in their study.^28^ Aspirin use (aOR: 0.84, 95% CI: 0.70‐1.01, *P* = 0.068) was not associated with a decreased risk of PDAC.^28^

 Additionally, Khalaf et al evaluated aspirin use and the risk of PDAC in 141,940 participants from the Health Professionals Follow-up Study (HPFS) and the Nurses’ Health Study (NHS).^15^ They defined 325-mg aspirin as the standard dose and 81-mg aspirin as the low dose. Also, regular aspirin users used aspirin (either standard or low-dose) at least twice per week on average.^15^ They also measured the pre-diagnosis plasma levels of salicylurate (a circulating metabolite of aspirin) in 396 nested PDAC cases and 784 controls from the HPFS, NHS, and Women’s Health Initiative-Observational Study cohorts.^15^ They did not find any association between regular aspirin use and incident PDAC in their pooled analysis of HPFS and NHS cohorts, and the pre-diagnosis level of salicylurate was not associated with the PDAC risk.^15^

 In contrast to large-scale cohort studies and clinical trials from the United States and Korea.^15,27,28^ two case-control studies from Shanghai, China, and Connecticut, USA (co-authored) supported the role of regular use of aspirin in reducing the incidence of PDAC.^14,29^ Besides, in a study from the UK, the effect of aspirin on mortality due to PDAC was only significant in patients receiving aspirin treatment for more than 7.5 years (HR: 0.28, 0.08–1.00, *P* = 0.04); its effect did not appear to increase at aspirin doses greater than 75 mg daily.^30^

 Two meta-analyses of observational studies examined the association between aspirin use and PDAC incidence and mortality in the past decade.^16,31^ In a meta-analysis involving 4748 PDAC cases and 252,025 healthy controls, Sun et al showed no significant association between aspirin use and the mortality risk of PDAC; however, the incidence of PDAC could slightly decrease with aspirin use (OR: 0.82, 95% CI: 0.68–0.98) with high heterogeneity (*P* = 0.001, I^2^ = 75.6%), and this effect was not dose-dependent.^16^ Moreover, analysis of six studies suggested that if the duration of aspirin use was < 5 years, it would not decrease the PDAC incidence.^16^ In another meta-analysis, Zhang et al reported a pooled estimate of decrease in the incidence of PDAC among aspirin users (OR: 0.77, 95% CI: 0.62 to 0.96).^31^ They also assessed the relationship between aspirin use and PDAC mortality in two cohort studies and did not observe a significant association.^31^

 The present results did not suggest that daily low-dose (80 mg) aspirin consumption could reduce the risk of PDAC development. The strengths of this study include accurate diagnosis of patients with PDAC by pathology, use of a prospective approach, large number of case subjects, and use of a valid questionnaire. On the other hand, the limitations of this study include: case–control format of the study which has recall bias, administration of only one dose of aspirin and the insufficient number of patients to evaluate the benefits of aspirin use for high-risk groups, such as diabetic patients. According to the review of several mentioned studies and the present research, a protective effect for aspirin uses and PDAC is not supported. Therefore, several large-scale clinical trials are recommended in different countries to obtain consistent results.
